# Brain Morphometry and the Neurobiology of Levodopa-Induced Dyskinesias: Current Knowledge and Future Potential for Translational Pre-Clinical Neuroimaging Studies

**DOI:** 10.3389/fneur.2014.00095

**Published:** 2014-06-12

**Authors:** Clare J. Finlay, Susan Duty, Anthony C. Vernon

**Affiliations:** ^1^Wolfson Centre for Age-related Diseases, King’s College London, London, UK; ^2^Department of Neuroscience, James Black Centre, Institute of Psychiatry, King’s College London, London, UK

**Keywords:** levodopa, magnetic resonance imaging, T_1_ relaxation, voxel-based morphometry, plasticity, prefrontal cortex

## Abstract

Dopamine replacement therapy in the form of levodopa results in a significant proportion of patients with Parkinson’s disease developing debilitating dyskinesia. This significantly complicates further treatment and negatively impacts patient quality of life. A greater understanding of the neurobiological mechanisms underlying levodopa-induced dyskinesia (LID) is therefore crucial to develop new treatments to prevent or mitigate LID. Such investigations in humans are largely confined to assessment of neurochemical and cerebrovascular blood flow changes using positron emission tomography and functional magnetic resonance imaging. However, recent evidence suggests that LID is associated with specific morphological changes in the frontal cortex and midbrain, detectable by structural MRI and voxel-based morphometry. Current human neuroimaging methods however lack sufficient resolution to reveal the biological mechanism driving these morphological changes at the cellular level. In contrast, there is a wealth of literature from well-established rodent models of LID documenting detailed *post-mortem* cellular and molecular measurements. The combination therefore of advanced neuroimaging methods and rodent LID models offers an exciting opportunity to bridge these currently disparate areas of research. To highlight this opportunity, in this mini-review, we provide an overview of the current clinical evidence for morphological changes in the brain associated with LID and identify potential cellular mechanisms as suggested from human and animal studies. We then suggest a framework for combining small animal MRI imaging with rodent models of LID, which may provide important mechanistic insights into the neurobiology of LID.

## Introduction

Parkinson’s disease (PD) is a multi-system neurodegenerative disorder that affects 1 in 100 people aged over 60 in the United Kingdom. The disease is characterized pathologically by the progressive degeneration of nigrostriatal dopamine (DA) containing neurons in the substantia nigra (1) and the accumulation of phosphorylated α-synuclein in Lewy bodies, ascending from the brain stem to the higher area association cortices as the disease progresses (2). The subsequent depletion of DA in the caudate and putamen of PD patients manifests itself as the classical triad of PD motor symptoms, akinesia, resting tremor, and rigidity/postural instability.

Some 44 years since its first use in the clinic (3), the first-line treatment for many PD patients to alleviate their motor symptoms remains pharmacological DA replacement with the DA precursor levodopa (l-DOPA) (4). Although most patients respond positively to l-DOPA treatment, after ~4–6 years of l-DOPA therapy, a significant proportion of patients (~40%) exhibit a decline in the therapeutic efficacy of l-DOPA and develop debilitating dyskinesias (5). This phenomenon, termed levodopa-induced dyskinesia (LID), is characterized by involuntary dystonic and/or choreic movements of the trunk, limbs, and face, most commonly when the plasma concentration of DA is high (“peak dose” dyskinesia) (5). The expression of LID severely limits the long-term clinical utility of l-DOPA in this sub-set of PD patients and thus significantly impacts on patient quality of life. As such, strategies to mitigate or prevent LID onset are the subject of intense research efforts to address this serious unmet medical need. In the clinic, these efforts are currently centered on modifying the timing, formulation, and mode of administration for l-DOPA. In particular, emphasis is placed on delaying l-DOPA treatment where possible by use of direct-acting DA receptor agonists (6) and if not possible, using continuous intestinal l-DOPA infusion rather than intermittent l-DOPA injections (7). Both approaches have met with some success in reducing the incidence and severity of LID. At the pre-clinical level, significant effort is being directed toward understanding the role of other neurotransmitter systems in LID, particularly the role of glutamate and serotonin, as well as the underlying molecular signaling pathways involved (8–10). Despite these efforts, the neural mechanisms underlying LID in PD remain obscure and the underlying neural correlates are not well understood. This presents a significant barrier to development of novel treatments (11).

In this article, we therefore examine current clinical evidence that suggests neuroanatomical changes in the brain are associated with LID, and the caveats associated with this. Second, we identify potential *post-mortem* cellular mechanisms as suggested from human and animal studies, which may explain these abnormalities. Thirdly, we outline the framework for combining small animal imaging with rodent models of LID, which may provide important mechanistic insights into the neurobiology of LID.

## Neural Correlates of Levodopa-Induced Dyskinesia: functional MRI Studies

Research efforts to unravel the neural correlates of LID in the clinic and in relevant animal models of LID are critical to address the gaps in our knowledge of LID pathogenesis (12, 13). A fruitful and translational strategy that can bridge clinical and pre-clinical studies to achieve this is the application of neuroimaging tools to both human patients and relevant animal models. In particular, the latter can provide a mechanistic framework to underpin neuroimaging observations in patients.

To date, such investigations have typically focused on the use of functional magnetic resonance imaging (fMRI) and positron emission tomography (PET). These studies have identified changes in brain network activity, metabolism and molecular changes related to LID onset and severity, as described elsewhere (14–17). In brief, it is clear from these studies that LID is associated with bi-directionally altered neuronal firing patterns between the basal ganglia and the neocortex, the net result of which is dis-inhibition of thalamo-cortical neurons, leading to over-activation of frontal cortical areas, particularly in the motor, pre-motor, and prefrontal cortices. These data have been confirmed in PD patients with LID using PET (14, 18), transcranial magnetic stimulation [TMS; Ref. (19)], task-based and resting state fMRI (17, 20, 21).

## Neuroanatomical Correlates of Levodopa-Induced Dyskinesia: Structural MRI Studies

Functional magnetic resonance imaging studies have revealed several important insights into LID pathophysiology. However, it is also true that human neuroimaging studies consistently demonstrate a linear relationship between the functional activity of the brain, assessed by fMRI and the shape, volume, or thickness of brain gray matter (22–24). These findings hold true for rodents as well (25, 26). This structure–function relationship is most likely driven by neuroanatomical remodeling at the cellular, synaptic (neuronal dendrite), or vascular level as a consequence of altered brain functional or metabolic activity (23, 27, 28). In other words, changes in brain function usually lead to or are concurrent with changes in the structure of the brain. Taking this into consideration, it is perhaps surprising that the use of structural MRI (sMRI) to probe whether there are neuroanatomical differences between patients with LID and those who are not dyskinetic has not been widely investigated.

In a recent study, the first of its kind to address this issue, Cerasa et al. (29) utilized optimized voxel-based morphometry (VBM) to analyze T_1_-weighted MR images from PD patients with LID (*n* = 36), non-dyskinetic PD patients (*n* = 36), and age- and sex-matched controls (*n* = 32). Compared to healthy controls, both dyskinetic and non-dyskinetic PD patients showed no significant differences in gray matter volume (GMV), somewhat consistent with other VBM findings in PD patients of a similar age and disease duration, although the sample size was small (29). However, when comparing dyskinetic versus non-dyskinetic PD patients directly, a significant *increase* in GMV was observed in the bilateral inferior frontal gyrus of the dyskinetic patients (29). This increase was negatively correlated to age at onset, such that the greatest increases in inferior frontal gyrus GMV were in LID expressing PD patients with younger age of onset (29). These data suggest a hypothesis that aberrant striato-frontal and/or thalamo-cortical neural plasticity associated with LID consequently leads to morphological remodeling of the prefrontal cortex (29), findings which have sparked an interesting debate (30, 31).

The normalization and smoothing processes inherent to the VBM pipeline may however lead to reduced sensitivity in assessing cortical pathology, since individual sulci and gyri cannot be accurately anatomically resolved (32, 33). As such, VBM therefore provides a mixed measure of gray matter reflecting two components, cortical surface area and cortical thickness. A direct measure of cortical thickness therefore represents a topographical measurement that might provide a more sensitive indicator of the integrity of the cytoarchitecture in the cortex (32, 33).

To address this, Cerasa et al. (34) used surface-based investigation of cortical thickness in PD patients with LID (*n* = 29), without LID (*n* = 30), and age- and sex-matched controls (*n* = 24). This analysis revealed a pronounced *increase* in the thickness of the right inferior frontal sulcus in the dyskinetic, as compared to non-dyskinetic patients (34). These data support their original VBM findings (29) and delineate with greater precision the anatomical abnormalities characterizing dyskinetic PD patients (34). A third study *combining* VBM and cortical thickness measurements in PD patients with LID (*n* = 33) or without LID (*n* = 33), stratified by their PD age-of-onset (< or >50 years of age), compared to age- and sex-matched healthy controls (*n* = 40) reveals further insights (35). Independent of the age of PD onset, dyskinetic patients were characterized by increased GMV and thickness in the inferior frontal cortex (35). Interestingly, early-onset PD patients with dyskinesia also demonstrated increased GMV in the substantia nigra and the red nucleus when compared to non-dyskinetic patients (35). In contrast, late-onset PD patients with dyskinesia were characterized by GMV increases in the supplementary motor area (SMA) only (35). Taken together, these data support the previous observations of anatomical abnormalities associated more generally with LID in the prefrontal cortex. Moreover, they demonstrate that different spatial patterns of brain abnormalities occur in patients with LID according to their age of PD onset. In particular, nigral pathology may be important in early-onset patients and in contrast, cortical pathology in late-onset patients (35).

## Levodopa, Gray Matter, and Magnetic Resonance Imaging Signal

When interpreting VBM results, it is important to eliminate artificial causes for differences between processed images that do not originate from genuine biological differences. In particular, MR image contrast between tissue classes on T_1_-weighted MR images is inversely proportional to the T_1_ relaxation time. Cerebrospinal fluid (CSF) is dark, reflecting a long T_1_; whereas the much shorter T_1_ of white matter renders it bright; and gray matter is intermediate between these. When the brain is segmented into these different tissue classes for volumetric analysis, VBM (and other automated techniques, including cortical thickness) utilizes voxel signal intensity profile (36). Each voxel has its own distinct intensity profile and there is substantial overlap in the voxel intensity histograms linked to gray and white matter. This renders precise tissue class segmentation difficult, particularly in the presence of partial volume effects, wherein a single voxel contains a mixture of tissue types (37). This is particularly common when a voxel spans distinct tissues, in cortical sulci (38). Such voxels are usually excluded or allocated to a particular tissue type on a probabilistic basis. As such, this segmentation process has the potential to go awry in the presence of unrecognized changes in voxel intensity profiles, leading to spurious volumetric findings (37). In other words, a reported volume change might in fact be an artifact of the signal acquisition and image analysis process (37).

This has recently been advanced as a biophysical explanation for the effects of lithium, a mood stabilizer, to apparently *increase* GMV (37). Whilst this notion is debated (39–41) and has yet to be explored in detail, it is certainly important and warrants attention (40, 41).

Interestingly, l-DOPA is associated with shortening of T_1_ (and T_2_) relaxation time *in vitro*, although this may be influenced by the presence of iron (42). This raises the possibility that changes in T_1_ in the human brain after l-DOPA administration may lead to an adjustment in the number of voxels that are attributed to gray matter. These would then be detected by VBM analysis as an apparent increase in GMV. Consistent with this hypothesis, VBM analysis reports an increase in voxels attributed to gray matter in the substantia nigra, ventral tegmental area, and subthalamic nucleus following *acute* l-DOPA administration in healthy volunteers (43). Unfortunately, this study did not include quantitative T_1_ parametric mapping to assess the impact of l-DOPA on T_1_ *in vivo*.

These findings following a single administration of l-DOPA to healthy people may lead to the suggestion differences in anatomical MRI data acquired from dyskinetic and non-dyskinetic PD patients (29, 34, 35), are driven by simple changes in signal intensity (grounded in increases in proton T_1_), which may be misinterpreted as a volume increase in the gray matter. Importantly, therefore, the gray matter volume increases reported in dyskinetic PD patients were detected only in dyskinetic patients compared to non-dyskinetic patients. All of these patients were receiving l-DOPA therapy at the time of scanning, with no significant difference in the duration of l-DOPA treatment (29, 34, 35).

Taken together, this does not support a general influence of l-DOPA on the MRI signal driving the observed results, since such an effect would be predicted to be present in both l-DOPA treated patient groups. The fact that the anatomical abnormalities are only present in the dyskinetic group strongly suggests these are inherently linked to the pathogenesis of LID. We note however that Lewis and colleagues report an accumulation of iron in the red nucleus of patients with l-DOPA-induced dyskinesia (44). Iron is paramagnetic and causes a reduction in both T_2_ and T_1_ relaxation time in brain regions where brain iron is deposited in the form of ferritin and hemosiderin (45). As such, whilst there is no evidence currently to suggest that neuroanatomical brain changes in dyskinetic PD patients are the result of an MR image artifact, quantitative T_1_ and T_2_ mapping may be recommended for future MRI studies in dyskinetic and non-dyskinetic patients. This would help to more accurately probe the exact origins of MRI signal changes in these groups. Alternatively, controlled animal studies may be important as MRI volume changes can be verified *post-mortem*, as we have shown previously (28, 39, 46, 47), and discussed later in this article.

## What is the Mechanism Underlying Neuroanatomical Change in Gray Matter Following Chronic l-DOPA Treatment?

Assuming that dyskinesia following l-DOPA treatment is associated with physical neuroanatomical changes in the brain, the next obvious issue is the identification of the underlying biological mechanism. However, neuroimaging measures are difficult to relate unambiguously to underlying biology (23). In particular, human neuroimaging studies cannot establish if the morphological abnormalities in the prefrontal cortex of dyskinetic PD patients are a cause or consequence of dyskinesia, following chronic l-DOPA treatment (29). One very plausible hypothesis is that these morphological changes reflect heightened activity within the neuronal circuitry implicated in LID pathogenesis (29). In other words, the detected pattern of brain abnormalities reflects altered neurobiological mechanisms central to the pathogenesis of LID. This hypothesis is supported by observations in hyperkinetic movement disorders such as dystonia. Indeed, dystonia is associated with exaggerated increases in GMV of specific brain regions involved in somatosensory processing, such as the basal ganglia, prefrontal cortex, and somatosensory cortex (35, 48). Furthermore, it is suggested that l-DOPA, when applied in a pulsatile and non-physiological manner may perturb the normal physiological mechanisms that mediate motor control (49). This process may lead to aberrant increases in synaptic plasticity, remodeling of neuronal synapses, and changes in the functional connectivity signature of brain activity within circuits responsible for motor control. At the cellular level, this is likely to manifest as increases in the size of neuronal dendritic arbors (50, 51). This phenomenon termed, “l-DOPA-maladaptive plasticity” may be critical to LID pathogenesis (29). Interestingly, this has also been suggested as an explanation for the incidence of tardive dyskinesia following chronic treatment with first generation antipsychotic drugs (52).

Evidence from recent studies using electroencephalography (EEG) and fMRI support this hypothesis. These reveal that brain activity and functional connectivity, defined as spontaneous, temporally coupled blood oxygen level dependent (BOLD) oscillations, is *decreased* in PD patients within the motor circuit in the cortex that receives dopaminergic innervation including the frontal, somatosensory, motor, and SMA (20, 21, 53–55). Furthermore, these studies confirm that acute and chronic l-DOPA treatment restores this lost connectivity with motor networks and increases neural activity in these brain regions (20, 21, 53–55). In an acute setting, this may explain the therapeutic effects of l-DOPA. However, with chronic treatment and the continued degeneration of the dopaminergic system, this could result in l-DOPA maladaptive plasticity leading to changes in neural arborization, and subsequently the observed neuroanatomical increases in the cortex of dyskinetic PD patients. Alternatively, such neuroanatomical enlargements could equally reflect a structural long-term consequence of the altered neural plasticity in these specific regions.

In reality however, MR phenomena are likely to be driven by several cellular processes; acting potentially in parallel in multiple cell types within the brain (23). Neuronal changes in gray matter as a cause or consequence of chronic l-DOPA treatment may include neurogenesis, synaptogenesis, and changes in neuronal morphology as discussed above. However, extra-neuronal changes may equally be responsible and these could include increases in glial cell size, morphology, number and additionally, angiogenesis. Indeed, the vasculature accounts for about 5% of gray matter (56). Glial cells (astrocytes, microglia, and oligodendrocytes) are believed to outnumber neurons by ~6 to 1, with varying ratios in different brain regions. Any of these cellular changes may influence MRI signals. Importantly, variations in neuronal, glial, and synaptic density may affect modalities sensitive to the proportion of cellular material versus extracellular space in a voxel, such as proton density imaging or relaxometry. Such features would therefore influence commonly used methods to assess gray matter change including VBM and cortical thickness that rely on image intensity boundaries in T_1_-weighted images (54).

Addressing this issue is problematic. In patients, one fruitful approach may be to conduct multi-modal neuroimaging in dyskinetic and non-dyskinetic PD patients to study the interrelationships between brain function, metabolism, and structure. This could include, but is not limited to, collection of resting state fMRI, sMRI and quantitative T_1_ mapping in the same session. Similarly, angiogenesis could be detected by techniques such as contrast-enhanced imaging of blood volume or perfusion imaging of cerebral blood flow (CBF). Recent advances in simultaneous PET–MRI, the feasibility of which has been demonstrated in rodents (57) and humans (58, 59) make this even more tractable and could provide unparalleled insights into the pathophysiology of LID.

Ultimately, *post-mortem* histological studies are required in order to make direct links between imaging measures and underlying mechanisms. For example, recent studies in our laboratory have established that neuroanatomical changes, including a reduction in the volume of the anterior cingulate cortex due to chronic antipsychotic drug treatment are not due to the loss of neurons or astrocytes (28). Similarly, in a very elegant study, mice subjected to different forms of maze training displayed volume increases in either the hippocampus (spatial maze) or striatum (cued maze), reflecting the distinct brain systems involved in these tasks (27). These MRI-derived measures of growth correlated with growth associated protein-43 (GAP-43) staining *post-mortem*, a marker for axonal growth cones, but not measures of neuronal size or number (27). These data suggest the observed MRI volume change reflected remodeling of neuronal processes, rather than neurogenesis (27). These data highlight the need to study relevant animal models to help unravel the neuroanatomical correlates of LID observed on MRI in patients.

## Animal Models of LID

Levodopa-induced dyskinesia can be modeled pre-clinically by recapitulating the conditions required for its development in humans, namely degeneration of the nigrostriatal tract followed by chronic exposure to l-DOPA. The primary dopaminergic cell loss models used for evaluation of dyskinesia are the 6-hydroxydopamine (6-OHDA) lesioned hemi-parkinsonian rat and the 1-methyl-4-phenyl-1,2,3,6-tetrahydropyridine (MPTP)-treated non-human primate (NHP). Repeated exposure of these denervated animals over several weeks to daily treatment with l-DOPA combined with a peripheral DOPA decarboxylase inhibitor such as benserazide or carbidopa, leads to development of abnormal involuntary movements (AIMs), which is considered an experimental proxy for human LID (13, 60–62). This can be scored according to a variety of ratings scales (63, 64). In the 6-OHDA-lesioned rat, AIMs manifest unilaterally as axial (twisting of the head, neck, and trunk), limb (repetitive or dystonic movements involving the forepaw and/or limb), and orolingual (vacuous chewing, tongue protrusion) phenomena on the side of the body contralateral to the lesion (60). In MPTP-treated NHPs, LID is bilaterally expressed and manifests as choreic and dystonic movements of the limbs, especially the lower limbs, and flicking of the fingers, trunk dystonias, and repetitive tongue protrusion (65). The MPTP–NHP model more accurately reflects the human expression of dyskinesia, but ethical and practical considerations mean that the 6-OHDA-lesioned rat model is a very valuable tool for pre-clinical research. Importantly, the mechanisms underlying the development of LID and AIMs appear to be common to both (13). Extensive research in both rat and NHP LID models, including studies to assess the potential of new anti-dyskinetic drug strategies, has identified a plethora of candidate mechanisms in specific brain regions, which may underlie the pathogenesis of LID (5, 66). Both striatal and extra-striatal systems are implicated, as described in brief below.

## Striatal Mechanisms Underlying LID

The role of DA in the striatum is to alter the response of medium spiny neurons (MSNs) in both the direct and indirect pathways to excitatory input from the corticostriatal pathway. The classical model of LID suggests the presence of high concentrations of exogenous DA (derived from l-DOPA) causes hyperactivation of the direct pathway (striatonigral) MSNs, which increases thalamocortical feedback and produces exaggerated motor function. Dyskinesia is therefore believed to primarily involve chronic over-activation of striatonigral MSNs (67, 68) and there is a wealth of evidence for a particular role of dopamine D1 receptors (D1R) in the development of LID in both patients and pre-clinical models (69–72). In reality the mechanisms involved are likely to be considerably more complex (73) and a role for the indirect pathway cannot be ruled out, especially as both D1R and dopamine D2 receptor (D2R) agonists can provoke dyskinesia in primed monkeys (74).

The striatonigral GABAergic projection is a point of convergence for, and is therefore modulated by, multiple neurotransmitter systems that may be pathologically altered in dyskinetic PD patients. The major input to the BG involves release of glutamate from corticostriatal neurones, and together with DA and the modulatory activity of other neurotransmitters such as serotonin (5-HT), this determines the activity of the output nuclei: the globus pallidus internus (GPi) and substantia nigra pars reticulata (SNr). There is evidence from animal models that this corticostriatal glutamate release is increased in LID (75, 76), alongside alterations in expression (69, 77–80), phosphorylation (81–83), and distribution (84, 85) of glutamate receptors, including NR1/NR2B NMDA receptors and metabotropic glutamate receptor 5 (mGlu_5_), that facilitate increased signaling across this synapse. Morphological alterations indicative of increased glutamatergic transmission are also present (86). This is borne out in human LID, where abnormal glutamatergic transmission has been described in the caudate, putamen, and motor cortex (87), alongside increased putaminal expression of NR1/NR2B NMDA receptors (88) and mGlu_5_ receptors (80). Activation of extrasynaptic NR2B-containing NMDA receptors has particularly been implicated in the development of LID (84).

The effect of this abnormal glutamatergic transmission may be compounded by the consequences of dysregulated release of DA from serotonergic terminals within the striatum (89), leading to abnormal activation of DA receptors. These receptors are expressed on striatonigral MSNs as well as in cortical dopaminergic systems, which have also been implicated in the pathophysiology of dyskinesia (90). There are some reports of altered D1R expression or trafficking in LID (70, 91), but evidence suggests that the key mechanism in dyskinesia is increased functional sensitivity of these receptors (92, 93).

Whatever the exact mechanism behind increased D1R signaling, stimulation of these receptors causes activation of the cyclic AMP (cAMP)/protein kinase A (PKA)/DARPP-32 (DA- and cAMP-regulated phosphoprotein, 32 kDa)/protein phosphatase 1 (PP-1) pathway and the mitogen activated protein kinase (MAPK) pathway, which culminates in phosphorylation of extracellular signal related kinase (ERK1/2) (94). This results in DNA modifications (95, 96) and increased expression of transcription factors, especially ΔFosB/FosB (97), which are indicative of long-term cellular adaptations.

Both NMDA and mGlu_5_ receptors are known to closely interact with D1R (81, 98) and with each other (99, 100), activating common downstream mediators such as PKA and ERK1/2 (101, 102). Therefore, the increased expression of these receptors alongside enhanced D1R signaling will co-operate to augment striatonigral signaling in LID. In addition, activation of D1R, in combination with enhanced activation of NMDA receptors by glutamate, leads to long-term potentiation-like phenomena. This may explain the lack of depotentiation seen in the dyskinetic versus non-dyskinetic denervated striatum (103), leading to an exaggerated response to normally irrelevant stimuli. The pathological overactivation of the direct pathway leads to GABA bursting in the SNr and GPi (71), thus disinhibiting thalamocortical feedback and leading to the hyperkinetic movements characteristic of LID.

Striatal glutamatergic and dopaminergic transmission can be modulated by several other neurotransmitters. For example, increased serotonergic innervation of the striatum, along with altered expression of several receptor subtypes (104, 105), has been demonstrated in animal and human LID (106, 107). Importantly, activation of serotonin 5-HT_1A_ receptors has been shown to reduce corticostriatal glutamate release (108, 109), and also negatively regulates release of DA as a false neurotransmitter from serotonergic terminals (110). Similarly the endocannabinoid system may play a role in LID as activation of CB_1_ receptors has been shown to negatively regulate corticostriatal glutamate release (111, 112) and also reduce D1R-mediated responses (113–115). Consequently, molecules such as serotonin receptor 5-HT_1A_ and 5-HT_1B_ agonists (116–120) and endocannabinoid receptor agonists (121–124) have shown anti-dyskinetic efficacy.

## Extra-Striatal Mechanisms of LID

As well as striatal alterations, there is also evidence from pharmacological studies suggesting that modulation of neurotransmission elsewhere in the BG and in areas of the cortex may also contribute to LID. Systemically active drugs could therefore produce anti-dyskinetic effects through actions at more than one key synapse. For example, antagonists of mGlu_5_, which are currently in clinical trials as anti-dyskinetic agents (125, 126), may exert their effects not only in the striatum but also in the subthalamic nucleus (127). Targeting of 5-HT_1A_ receptors in the subthalamic nucleus (128) or primary motor cortex (129) also attenuates dyskinesia, as does activation of 5-HT_1B_ receptors (130, 131), which are not only present in the striatum but also on GABAergic MSNs terminating in the SNr, where their activation can inhibit GABA release (132). As well as the striatal actions already mentioned, another potential mechanism to explain the efficacy of CB_1_ agonists is potentiation of striatopallidal signaling via inhibition of GABA reuptake (121), which would help to rebalance a hyperactivation of striatonigral signaling. Opioid signaling, which is known to be altered in LID (133–135), can modulate transmitter release at several synapses within the BG, for example inhibition of striatopallidal GABA release (136), and inhibition of glutamate and GABA release into the SNr (137). Targeting several opioid receptor subtypes has shown anti-dyskinetic efficacy (138–141), but their role is complex and the effects of opioid-targeted approaches may be dose-dependent (137).

## Future Directions: A Translational Road Map to Bridge Animal Mechanistic Studies with Brain Structural Imaging to Identify the Morphological Correlates of LID

**Figure 1:**
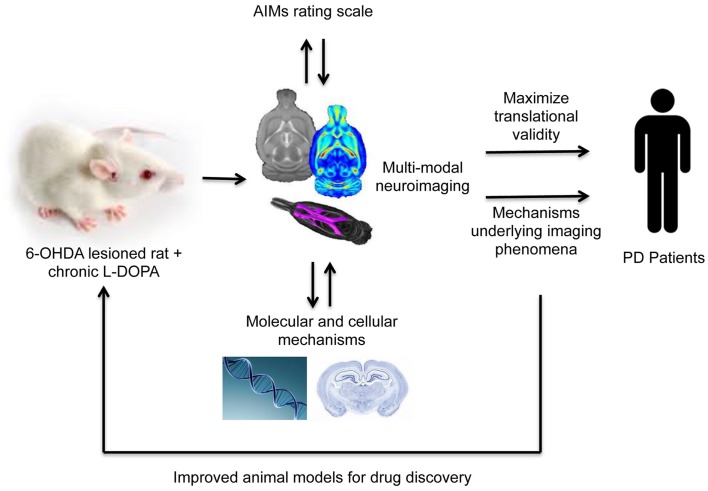
**A translational methodological framework for the combination of rodent models of LID (or these could be non-human primates) with multi-modal imaging, behavior and post-mortem cellular, or molecular analysis to elucidate the mechanisms underlying imaging phenomena associated with levodopa dyskinesia observed in human Parkinson’s disease patients**.

These maladaptive plastic changes described above in the striatum and extra-striatal regions may well underlie the morphological changes associated with LID described in humans. Combining well-validated rat and potentially, NHP, models of LID with advanced non-invasive animal MR imaging methods therefore offers an exciting opportunity to integrate currently disparate areas of research and help explain the MR imaging phenomena observed in dyskinetic patients (31) (Figure 1). This approach is advantageous for three reasons. First, rodents and primates allow one to assess the precise effects of drug treatment (in this case, l-DOPA) on brain structure and function, disentangled from potential confounding factors present in patient samples. The proof-of-concept for this approach has been recently demonstrated in our laboratory in characterizing the impact of chronic antipsychotic drug treatment on rat brain morphometry (28, 39, 142). Second, the use of MRI/PET (clinically comparable technology) permits the collection of parallel data read-outs in rodents, primates, and humans, maximizing the possibility for translation of basic findings to the clinic. Thirdly, and most importantly, as we have seen in the preceding section, in animals one can measure neurochemical, biochemical, cellular, and molecular aspects of brain structure and function in ways that are impossible in human subjects. Thus neuroimaging and neuropathology may be bridged to identify the biological mechanisms underlying MRI phenomena.

A number of elegant functional imaging studies using small animal micro-PET technology have begun to address this. Studies using PET in 6-OHDA lesioned rats chronically treated with l-DOPA and displaying severe AIMs (the rodent LID phenotype) display regional increases in CBF (measured using [14C]-iodoantipyrine uptake), increases in regional cerebral glucose utilization (rCGU; measured using [14C]-2-deoxyglucose uptake) and DA release (measured using displacement of [11C]-raclopride binding potential), consistent with similar findings in PD patients (143–145). To date however, no studies have assessed the impact of l-DOPA treatment on brain morphometry or relaxation time in either rodent or primates, using advanced structural MR imaging methods. This approach offers a unique opportunity to answer a number of outstanding questions in the emerging field of neuroanatomical alterations linked to l-DOPA and LID pathogenesis. Multi-modal imaging approaches, to study the interrelationships between brain anatomy [sMRI, Diffusion Tensor Imaging (DTI)] metabolism [2-dexoyglucose, ^1^H-magnetic resonance spectroscopy (MRS)] and fMRI may be of particular relevance. These imaging findings may then serve as a “roadmap” to guide follow-up, region-specific *post-mortem* investigation of the candidate mechanisms already identified in these models, to help explain the imaging phenomena. Excitingly, such studies are now underway in our laboratory.

Combining experimental models of LID with clinically comparable technology may also be particularly important for the assessment of novel anti-dyskinetic drugs, such as antagonists of mGlu_5_. Indeed, as previously stated, the use of clinically comparable technology (MRI) to conduct parallel assessments in experimental animals and humans is likely to accelerate translation of basic findings to the clinic. Neuroimaging tools may therefore play a critical role in future studies evaluating not only target engagement, but also drug efficacy in models of LID, as evidenced by recent studies using PET (143, 144, 146). No studies as yet have employed MRI methods, but the potential for application of this technology is apparent.

## Conclusion

Recent human sMRI studies in PD patients with dyskinesia have suggested the presence of neuroanatomical changes in specific brain regions, particularly the frontal cortex, which may have relevance to the pathogenesis of dyskinesia. It is currently unclear from these studies whether these abnormalities are the cause, or consequence of dyskinesia. Furthermore, it is unclear if these reflect genuine neuroanatomical changes in shape, thickness, or volume of gray matter, or whether these can be explained by a biophysical hypothesis relating to l-DOPA, as recently described for the effects of lithium. Accepting this caveat and presuming these changes to be genuine structural differences in gray matter, the biological mechanism underlying these changes remain unknown, but may be rooted in maladaptive neuronal plasticity, leading to remodeling of synapses and dendrites on neurons and glia alike in the dyskinetic brain. However, a plethora of data for candidate mechanisms underlying the pathophysiology of LID exists from well-validated rodent and NHP pre-clinical models of LID, which display excellent construct, face, and predictive validity to human LID. The combination of these models with advanced, multi-modal small animal MR imaging technology therefore offers a unique opportunity to validate the presence of neuroanatomical changes associated with LID. Furthermore, this will be an important step to bridge neuroimaging and neuropathology to link candidate mechanisms derived from animal models with neuroimaging phenomena in dyskinetic PD patients. Ultimately, this will accelerate our understanding of LID pathogenesis and aid the discovery and evaluation of novel anti-dyskinetic drug treatments.

## Conflict of Interest Statement

The authors declare that the research was conducted in the absence of any commercial or financial relationships that could be construed as a potential conflict of interest.
